# Stabilization of the Virulence Plasmid pSLT of *Salmonella* Typhimurium by Three Maintenance Systems and Its Evaluation by Using a New Stability Test

**DOI:** 10.3389/fmolb.2016.00066

**Published:** 2016-10-17

**Authors:** Damián Lobato-Márquez, Laura Molina-García, Inma Moreno-Córdoba, Francisco García-del Portillo, Ramón Díaz-Orejas

**Affiliations:** ^1^Section of Microbiology, Department of Medicine, Centre for Molecular Bacteriology and Infection, Imperial College LondonLondon, UK; ^2^Department of Cell and Developmental Biology, University College LondonLondon, UK; ^3^Departamento de Microbiología Molecular y Biología de las Infecciones, Centro de Investigaciones Biológicas-Spanish National Research CouncilMadrid, Spain; ^4^Departamento de Biotecnología Microbiana, Centro Nacional de Biotecnología-Spanish National Research CouncilMadrid, Spain

**Keywords:** virulence plasmid, toxin-antitoxin, plasmid stability, transcriptional regulation, *Salmonella* Typhimurium

## Abstract

Certain *Salmonella enterica* serovars belonging to subspecies I carry low-copy-number virulence plasmids of variable size (50–90 kb). All of these plasmids share the *spv* operon, which is important for systemic infection. Virulence plasmids are present at low copy numbers. Few copies reduce metabolic burden but suppose a risk of plasmid loss during bacterial division. This drawback is counterbalanced by maintenance modules that ensure plasmid stability, including partition systems and toxin-antitoxin (TA) loci. The low-copy number virulence pSLT plasmid of *Salmonella enterica* serovar Typhimurium encodes three auxiliary maintenance systems: one partition system (*parAB*) and two TA systems (*ccdAB*_ST_ and *vapBC2*_ST_). The TA module *ccdAB*_ST_ has previously been shown to contribute to pSLT plasmid stability and *vapBC2*_ST_ to bacterial virulence. Here we describe a novel assay to measure plasmid stability based on the selection of plasmid-free cells following elimination of plasmid-containing cells by ParE toxin, a DNA gyrase inhibitor. Using this new maintenance assay we confirmed a crucial role of *parAB* in pSLT maintenance. We also showed that *vapBC2*_ST_, in addition to contribute to bacterial virulence, is important for plasmid stability. We have previously shown that *ccdAB*_ST_ encodes an inactive CcdB_ST_ toxin. Using our new stability assay we monitored the contribution to plasmid stability of a *ccdAB*_ST_ variant containing a single mutation (R99W) that restores the toxicity of CcdB_ST_. The “activation” of CcdB_ST_ (R99W) did not increase pSLT stability by *ccdAB*_ST_. In contrast, *ccdAB*_ST_ behaves as a canonical type II TA system in terms of transcriptional regulation. Of interest, *ccdAB*_ST_ was shown to control the expression of a polycistronic operon in the pSLT plasmid. Collectively, these results show that the contribution of the CcdB_ST_ toxin to pSLT plasmid stability may depend on its role as a co-repressor in coordination with CcdA_ST_ antitoxin more than on its toxic activity.

## Introduction

During evolution bacterial pathogens acquire new genes dedicated to manipulate host processes. Many of these pathogen functions are encoded by chromosomal genes. Others, however, can be encoded by genes present in mobile genetic elements such as virulence plasmids. Horizontal transfer of these mobile genetic components has shaped the host adaptation strategies in several bacterial pathogens (Jackson et al., 2011). The presence of a virulence gene in a mobile element also facilitates its rapid acquisition or loss under distinct selective pressures. Enteric bacteria such as *Escherichia coli, Shigella* spp. and *Salmonella enterica*, frequently carry virulence genes in large transmissible low-copy-number plasmids (Sasakawa et al., 1986; Makino et al., 1988; Gulig et al., 1993). The *S. enterica* species are facultative intracellular bacteria that cause disease ranging from self-limiting gastroenteritis to more severe systemic infections (Rivera-Chávez and Bäumler, 2015). *S. enterica* subdivides into seven subspecies (I, II, IIIa, IIIb, IV, VI, and VII) (Tindall et al., 2005; Grimont and Weill, 2007) and subspecies I includes more than 2500 serovars (Grimont and Weill, 2007). Most of these serovars have adapted to infect warm-blooded hosts. One of the most extensively studied serovars of subspecies I is Typhimurium, which infects both humans and livestock. Serovar Typhimurium, together with a few other serovars of subspecies I, possesses a virulence plasmid (Jones et al., 1982). These plasmids have a variable size of 50–90 kb and share common features such as low copy number (1-2 plasmids per chromosome), a similar *repC* replicon (similar to the *repFIB* family) and a conserved set of virulence genes encoding toxins and fimbrial proteins (including *spv* and *pef* operons) (Bäumler et al., 1998; Rotger and Casadesús, 1999). The low copy number of the *S*. Typhimurium virulence plasmid (also called pSLT) could theoretically compromise its heritability to daughter cells during cell division. Despite this, pSLT is extremely stable in the host with ~10^−7^ segregants per cell generation, in a similar rate to that observed for other low-copy-number plasmids such as F and P1 (Austin and Abeles, 1983; Kline, 1985; Tinge and Curtiss, 1990a). Low-copy-number plasmids carry maintenance modules such as partition systems and toxin-antitoxin (TA) systems that ensure their proper segregation to nascent cells (Million-Weaver and Camps, 2014). Partition systems significantly increase the stability of plasmids by ensuring segregation of one copy of the plasmid to each sibling cell (Ebersbach and Gerdes, 2005). On the other hand, TA modules are typically bicistronic operons that encode an unstable antitoxin and a stable toxin (Chan et al., 2016; Lobato-Márquez et al., 2016). As a consequence of their different stabilities, antitoxin must be continuously produced to efficiently neutralize its cognate toxin (Gerdes et al., 1986). However, if the TA-encoding plasmid is lost, the antitoxin cannot be replenished and the free toxin eliminates or reduces the growth of daughter cells thus diluting plasmid-free cells in the population (Yamaguchi and Inouye, 2011). This phenomenon is called post-segregational killing (Gerdes et al., 1986).

Classically, plasmid stability has been measured using antibiotic-resistance plasmid derivates. Cells harboring the studied plasmid are positively selected in the presence of the selection antibiotic and those that have lost the plasmid are killed (Gerdes et al., 1985; del Solar et al., 1987). The main drawback of this technique is its sensitivity. Highly stable plasmids such as *S*. Typhimurium pSLT are below the sensitivity range of these assays. To solve this problem other methods relying in the direct selection of plasmid-free cells have been developed; for instance, the one based on the *tetAR*-chlortetracycline system (Bochner et al., 1980; Maloy and Nunn, 1981). The *tetA* gene encodes a protein which resides in the cytoplasmic membrane and prevents cellular accumulation of tetracycline, thereby conferring resistance (Reyrat et al., 1998). However, TetA location in the bacterial membrane also causes the cell to become hypersensitive to lipophilic chelators such as fusaric or quinalic acids (Bochner et al., 1980). Therefore, it is possible to select those cells that have lost the *tetAR* cassette. Inserted in a plasmid, the *tetAR* cassette can be used to select plasmid-free cells in special agar plates (Bochner-Maloy) containing fusaric acid (García-Quintanilla et al., 2006). Limitations of this method include poor reproducibility and the frequent occurrence of false positives (Li et al., 2013). Here, we have developed a novel, highly sensitive stability assay based on the negative selection of plasmid-containing cells. This assay is based on a cassette containing the ParE toxin-encoding gene of the *parDE* TA system and a kanamycin resistance gene (*aph*). ParE toxin targets DNA gyrase, blocks DNA replication and induces DNA breaks leading to cell death (Jiang et al., 2002). In our system ParE synthesis is controlled by a rhamnose-inducible promoter (*P*_*parE*_) (Maisonneuve et al., 2011). Once the *aph*-*parE* cassette has been inserted in the plasmid of interest and upon induction of *P*_*parE*_, only plasmid-free cells survive. Using this new tool we studied the contribution of the three main maintenance modules of the pSLT virulence plasmid of *S*. Typhimurium: the *parAB* partition system (Tinge and Curtiss, 1990b) and the *ccdAB*_ST_ and *vapBC2*_ST_ TA loci (Lobato-Márquez et al., 2015). We show that *vapBC2*_ST_ TA module, which we recently demonstrated to be important to *S*. Typhimurium survival during non-phagocytic cells infection (Lobato-Márquez et al., 2015, 2016), also stabilizes pSLT plasmid. We show that the *ccdAB*_ST_ TA system, known to impact pSLT heritability and encoding an inactive toxin (García-Quintanilla et al., 2006; Lobato-Márquez et al., 2015), conserves its TA transcriptional regulatory activity. Of interest, the *ccdAB*_ST_ operon extends beyond the toxin gene including four additional open reading frames. Moreover, CcdAB_ST_ TA complexes influence expression of downstream genes. We also demonstrate that stability of pSLT plasmid is not affected by a mutation (R99W) that restores CcdB_ST_ toxicity. We propose that the contribution of *ccdAB*_ST_ to pSLT stability could be related to the regulatory activity of CcdA_ST_-CcdB_ST_ complexes rather than to a post-segregational killing effect mediated only by CcdB_ST_ toxicity.

## Results

### Development of a new assay to measure plasmid stability

**Figure 1:**
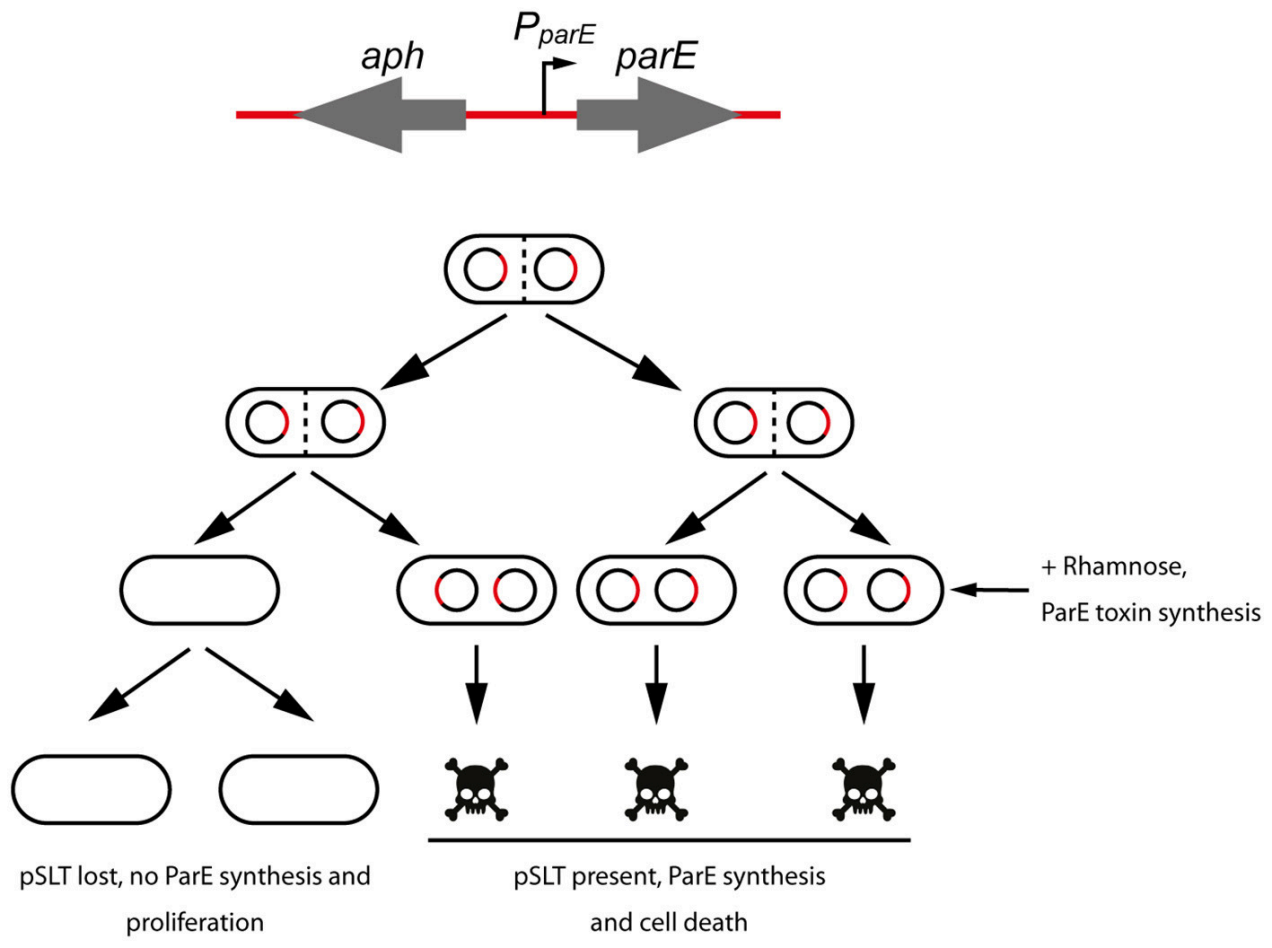
**Scheme of the method used to measure plasmid stability of *S*. Typhimurium pSLT derivates**. A cassette containing a kanamycin resistance gene (*aph*) and the DNA gyrase inhibitor ParE-encoding gene was used to disrupt genes of interest **(top)**. Grown cultures were plated in rhamnose-containing agar plates and synthesis of ParE toxin was induced. Cells that kept pSLT plasmid and therefore *aph*-*parE* cassette, were selectively killed **(bottom)**.

Due to the recognized problems of the *tetAR*-chlortetracycline method to measure plasmid stability, we decided to develop a novel negative selection method to measure the contribution to stability of the different maintenance modules encoded in pSLT plasmid (Figure 1). We took advantage of an *aph*-*parE* cassette of the pKD267 plasmid (Maisonneuve et al., 2011). This cassette carries a kanamycin resistance gene (*aph*) and the *parE* gene, which encodes the toxin of the *parDE* TA system. ParE toxin interacts with and blocks the DNA gyrase, causing inhibition of DNA synthesis, induction of breaks and nicks in the DNA and finally cell death (Jiang et al., 2002). In the *aph*-*parE* cassette, previously used for chromosomal scarless deletions (Maisonneuve et al., 2011; Lobato-Márquez et al., 2015), the toxin-encoding *parE* gene is controlled by a rhamnose-inducible promoter. Thus, when rhamnose is present as the only carbon source in the medium, ParE is synthesized and the cell is killed (Figure 1). Using *aph*-*parE* cassette to disrupt the maintenance modules of pSLT plasmid we could select plasmid-free bacteria. To distinguish plasmid curing from other events causing rhamnose resistance (e.g., mutations in *P*_*parE*_ promoter or *parE* gene), we took advantage of the kanamycin resistance gene also present in the *aph*-*parE* cassette. The resulting pSLT plasmid derivates were thus tagged with two different markers.

### *parAB* partition system and *vapBC2*_ST_ promote stability of *S*. Typhimurium pSLT plasmid

**Figure 2:**
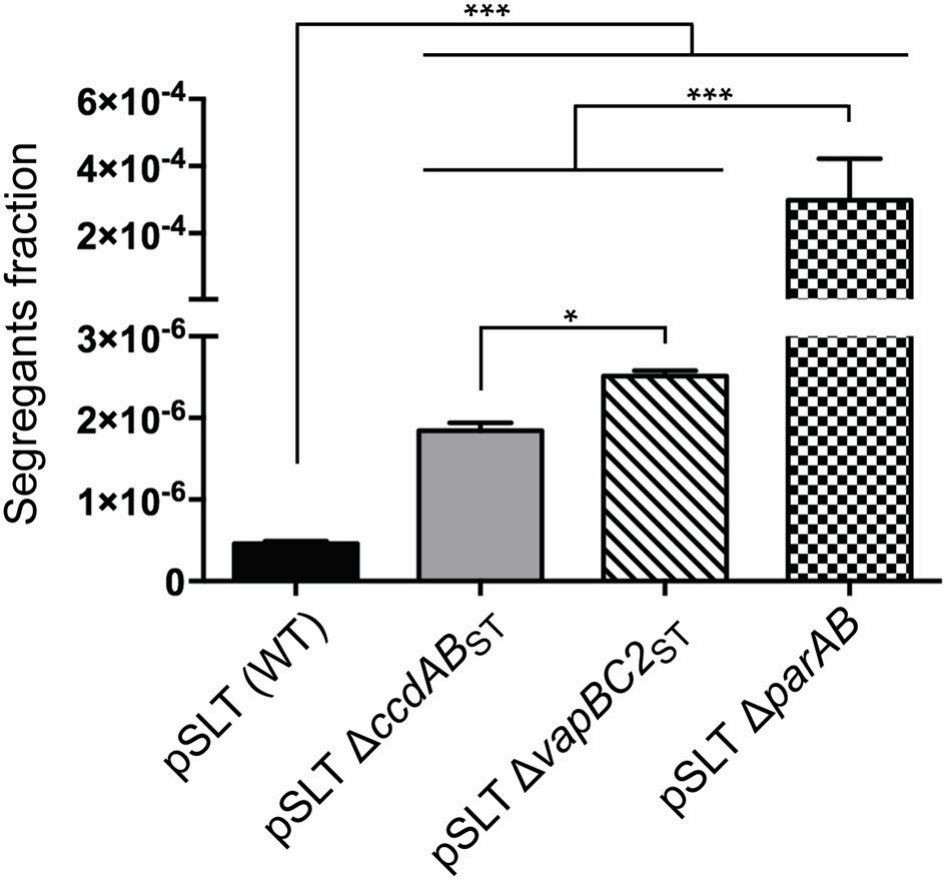
**Plasmid stability assays of *S*. Typhimurium pSLT derivates**. Segregants fraction measurement of pSLT plasmid comparing bacteria bearing the following plasmids: (i) wild type pSLT, (ii) pSLT lacking *ccdAB*_ST_ TA module, (iii) pSLT lacking *vapBC2*_ST_ TA system, and (iv) pSLT lacking *parAB* partition system. Disruption of all maintenance modules significantly decreased pSLT stability. The fraction of segregants was determined dividing the number of colony forming units grown in M9-rhamnose-agar plates (plasmid-free bacteria) by total bacteria grown in LB-agar plates. Data represent the means and standard deviations from five independent experiments. ^*^P < 0.05; ^***^P < 0.001 by one-way ANOVA and Tukey's multiple comparison post-test.

Previous studies proposed two important regions involved in *S*. Typhimurium pSLT plasmid stability: the *parAB* partition system and the TA module *ccdAB*_ST_ (Tinge and Curtiss, 1990b; García-Quintanilla et al., 2006). Additionally, we identified another TA system, called *vapBC2*_ST_, encoded within the *trbH* gene (Lobato-Márquez et al., 2015) and homologous to the *mvpAT* locus encoded in the virulence plasmid of *Shigella flexneri* (Sayeed et al., 2000). We reported that *vapBC2*_ST_ promotes *Salmonella* survival inside infected host cells. We now evaluated if similarly to *ccdAB*_ST_, and to other plasmidic TA loci, *vapBC2*_ST_ may play a role in pSLT stability. Additionally, to test the sensitivity and the reproducibility of our method, we reevaluated the contribution of *parAB* and *ccdAB*_ST_ using the new stability assay. We compared pSLT plasmids derivates lacking *parAB, ccdAB*_ST_ or *vapBC2*_ST_ with an isogenic strain in which *aph*-*parE* cassette was inserted in the gene *spvA*, which was previously shown to be innocuous for the stability of pSLT (García-Quintanilla et al., 2006). Stability assays demonstrated that disruption of *vapBC2*_ST_ TA system resulted in a 5.5 ± 0.1 fold increase in the fraction of segregants after ~10 generations of growth without selection pressure (Figure 2). This increase was more important than in the case of the pSLT derivate lacking *ccdAB*_ST_ (4 ± 0.2) under the same growth conditions (Figure 2). In accordance to previous studies, disruption of *parAB* or *ccdAB*_ST_ decreased pSLT stability (Tinge and Curtiss, 1990a; García-Quintanilla et al., 2006). The *parAB* partition system stabilizes pSLT plasmid 119 ± 3 and 163 ± 9 fold more efficiently than the *vapBC2*_ST_ or *ccdAB*_ST_ TA systems, respectively (Figure 2). Moreover, the pSLT wild type plasmid was 650.1 ± 190.2 fold more stable than pSLT lacking *parAB* (Figure 2). These data strongly suggested that *parAB* is the main contributor to pSLT heritability. However, *ccdAB*_ST_ and *vapBC*_ST_ TA systems showed a moderate contribution to pSLT stability. Together, these results demonstrated the potential of this new stability assay to determine accurately plasmid lost rates, being able to detect ~1 segregant in 2·10^6^ bacteria. Moreover, we demonstrated that *vapBC2*_ST_, apart from its contribution to *S*. Typhimurium virulence, also mediates pSLT heritability.

### CcdB_ST_ toxicity is not required for *ccdAB*_ST_-mediated stability of pSLT

**Figure 3:**
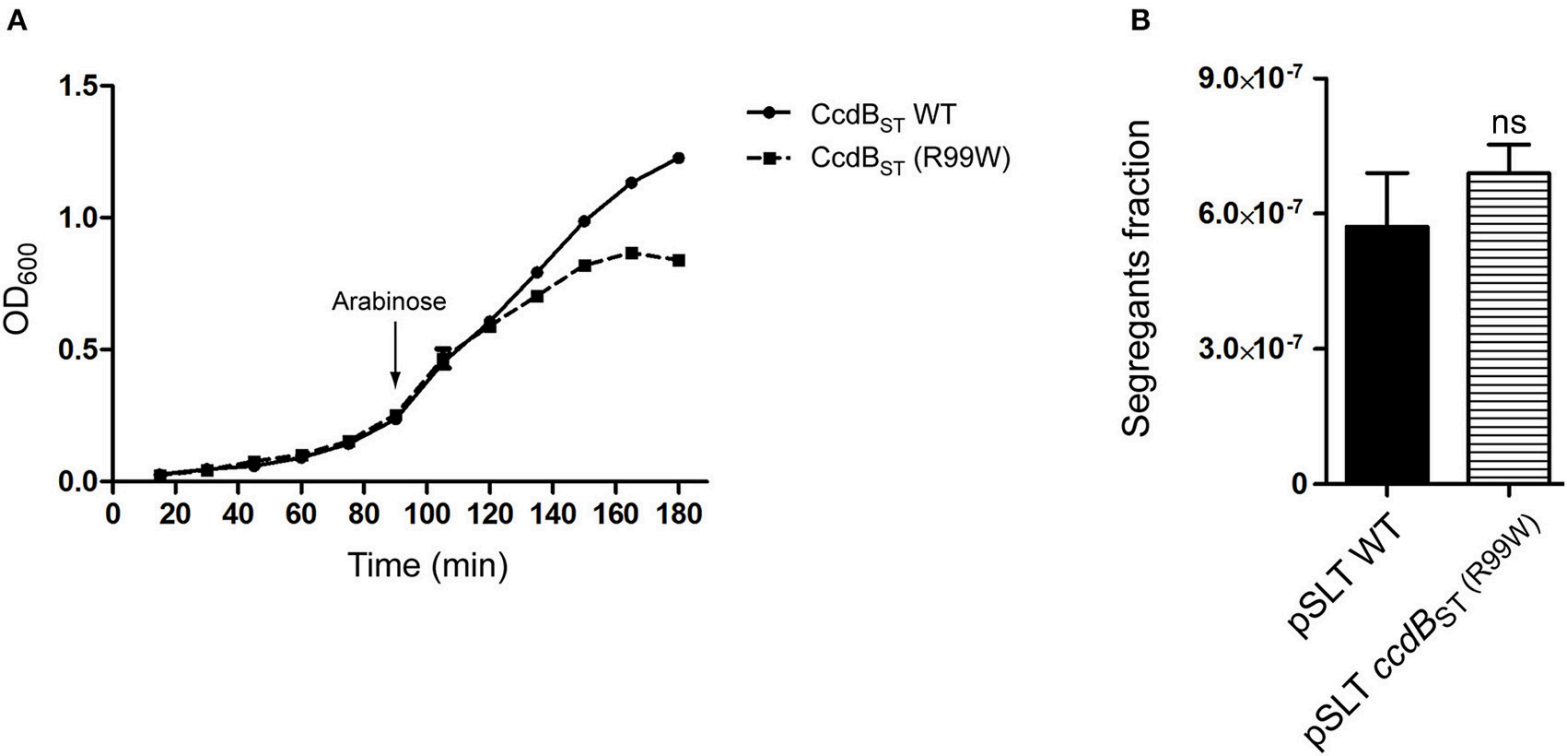
**The toxic activity of CcdB_ST_ is dispensable for the CcdAB_ST_-mediated pSLT stability. (A)** Growth curves of *S*. Typhimurium strains expressing wild type non-active or CcdB_ST_ (R99W) toxic proteins. Bacteria were grown at 37°C with shaking in LB medium. The expression of the *ccdB*_ST_ (R99W) toxin-encoding gene ceased bacterial growth. The arrow indicates the time point (90 min) at which CcdB_ST_ synthesis was induced by arabinose addition. **(B)** Segregants fraction measurement of pSLT plasmid comparing pSLT wild type and a pSLT variant harboring the toxic version *ccdB*_ST_ (R99W). Data represent the means and standard deviations from five independent experiments. Data were compared using Student's *T*-test. ns, non significant.

Our stability assays agreed with a previous study reporting contribution of *ccdAB*_ST_ to pSLT plasmid stability (García-Quintanilla et al., 2006). We have recently demonstrated that CcdB_ST_ toxin of *S*. Typhimurium is not functional due to an amino acid substitution in the position 99 (W99R) (Lobato-Márquez et al., 2015). This residue is essential for the binding of CcdB to the subunit A of DNA Gyrase (GyrA) (Bahassi et al., 1995; Dao-Thi et al., 2005). The lack of toxic activity in CcdB_ST_ was further confirmed in liquid cultures of *S*. Typhimurium expressing either wild type (inactive) or active (R99W) versions of CcdB_ST_ (Figure 3A).

In the F plasmid, the ortholog TA module *ccdAB* contributes to plasmid stability through a mechanism called “post-segregational killing” (Gerdes et al., 1986). Cells that do not inherit a copy of the TA-encoding plasmid cannot synthesize new antitoxin, leading the toxin free to kill or reduce the growth of plasmid-free cells (Van Melderen et al., 1994). We asked whether in *S*. Typhimurium the toxicity of CcdB_ST_ could be important for pSLT stability. To test this hypothesis, we carried out stability assays using a pSLT plasmid in which de non-functional *ccdB*_ST_ was substituted by an activated *ccdB*_ST_ (R99W) variant. Stability assays showed no differences between the pSLT plasmid derivates containing wild type *ccdB*_ST_ or the toxic version *ccdB*_ST_ (R99W), suggesting that CcdB_ST_ toxicity is dispensable for *ccdAB*_ST_-dependent stability (Figure 3B). Due to the ability of CcdAB_ST_ TA system to stabilize pSLT plasmid independently of CcdB_ST_ toxicity, we characterized the *ccdAB*_ST_ operon in more detail.

### The non-functional TA system *ccdAB*_ST_ of *S*. Typhimurium conserves transcriptional regulatory activity

**Figure 4:**
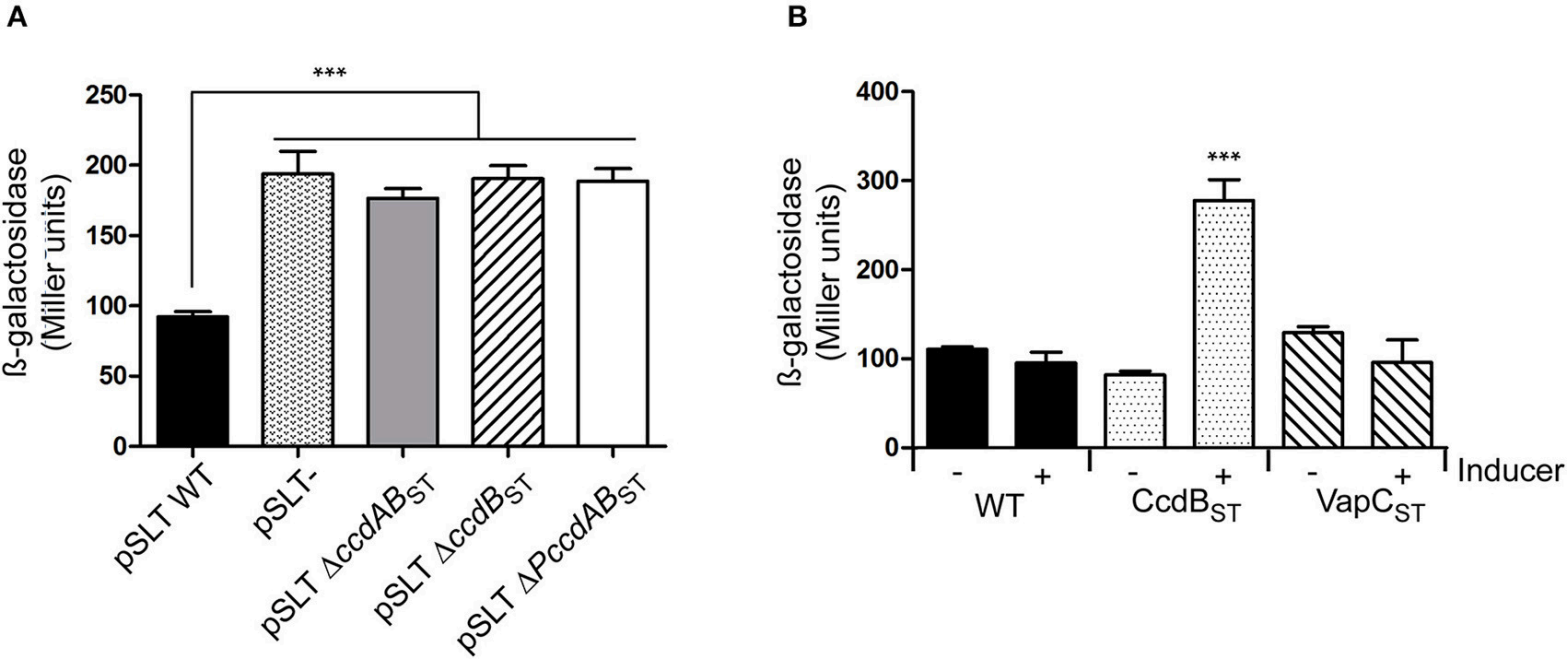
**Transcriptional regulation of the *ccdAB*_ST_ TA system**. The 300 bp region upstream to *ccdA*_ST_ was cloned as a transcriptional fusion with a promoter-less *lacZ* reporter gene in plasmid pMP220. **(A)** β-galactosidase was measured in different genetic backgrounds of *S*. Typhimurium SV5015 transformed with pPccdAB-lacZ: (i) pSLT wild type, (ii) a strain cured of pSLT, (iii) a pSLT plasmid deficient for *ccdAB*_ST_ TA system (including the promoter of the system), (iv) a pSLT lacking the toxin *ccdB*_ST_ gene, and (v) a pSLT in which the *PccdAB*_ST_ promoter was eliminated. Absence of TA complexes releases the CcdAB_ST_ transcriptional repression, resulting in increasing β-galactosidase activity **(B)** Wild type *S*. Typhimurium SV5015 was transformed with pPccdAB-lacZ and either a plasmid expressing the non-functional copies of CcdB_ST_ or VapC_ST_ to further measure β-galactosidase activity. Cultures were grown to OD_600_ of 0.3 and then expression of *ccdB*_ST_ or *vapC*_ST_ genes was induced by adding 0.3% arabinose during 1 h. Excess of CcdB_ST_ specifically shows conditional cooperativity effect as its overexpression derepresses transcription at *PccdAB*_ST_ promoter of PSLT plasmid. Data represent the means and standard deviations from four independent experiments. ^***^P < 0.001 by one-way ANOVA.

We tested if the type II TA module *ccdAB*_ST_ of *S*. Typhimurium behaves as a *bona fide* TA system in terms of transcriptional regulation. In the F plasmid the antitoxin CcdA of the *ccdAB* ortholog acts as a transcriptional repressor and the toxin enhances the repressor activity when TA complexes are formed in a proper stoichiometry (Tam and Kline, 1989; Salmon et al., 1994). Mutations in the last three amino acids of the CcdB toxin in the F plasmid eliminate its toxicity while maintain its regulatory activity (Bahassi et al., 1995). To test the transcriptional activity of *S*. Typhimurium *ccdAB*_ST_, we fused the promoter of the TA system (*PccdAB*_ST_) to a promoter-less *lacZ* reporter gene. We measured β-galactosidase activity in the following genetic backgrounds: (i) pSLT wild type, (ii) pSLT plasmid cured, (iii) pSLT deficient for *ccdB*_ST_ gene, (iv) pSLT deficient for *ccdAB*_ST_ operon, and (v) pSLT only lacking promoter *PccdAB*_ST_. β-galactosidase assays demonstrated that *ccdAB*_ST_ TA module behaves as a classical type II TA system. When the whole system is present (wild type background), transcription of the operon is repressed. However, this repression is lost in the absence of CcdAB_ST_ repressor complexes due to the lost of either *ccdB*_ST_ or *ccdAB*_ST_ (Figure 4A). Interestingly, we did not observe differences in β-galactosidase activity when the system lacked only the toxin *ccdB*_ST_ or the whole operon arguing for an important role of CcdB_ST_ in transcriptional regulation.

In many type II TA modules, transcriptional regulation relies on the toxin:antitoxin ratio. Thus, an excess of antitoxin results in TA complexes that are efficient repressors; however, when the number of toxin molecules increases, the stoichiometry of the complex changes and repression is relieved. This regulation feature is termed “conditional cooperativity” (Overgaard et al., 2008). Taking advantage of the inactive CcdB_ST_ toxin, we analyzed the conditional cooperativity phenomenon in the *ccdAB*_ST_ TA module of pSLT plasmid by supplying *in trans* an extra dose of the inactive CcdB_ST_ toxin. We employed a plasmid that contains inactive *ccdB*_ST_ gene controlled by an arabinose-inducible promoter. To discard unspecific effects derived from protein over-production, the same experiment was carried out with the unrelated non-toxic VapC_ST_ toxin encoded in the *S*. Typhimurium chromosome (Lobato-Márquez et al., 2015). Upon arabinose addition, we specifically observed an increased transcriptional activity of the *PccdAB*_ST_ promoter following CcdB_ST_ but not VapC_ST_ production (Figure 4B). These data demonstrate that the *ccdAB*_ST_ TA system responds to conditional cooperativity.

### *ccdAB*_ST_ of *S*. Typhimurium pSLT plasmid conforms a six-gene polycistronic operon

**Figure 5:**
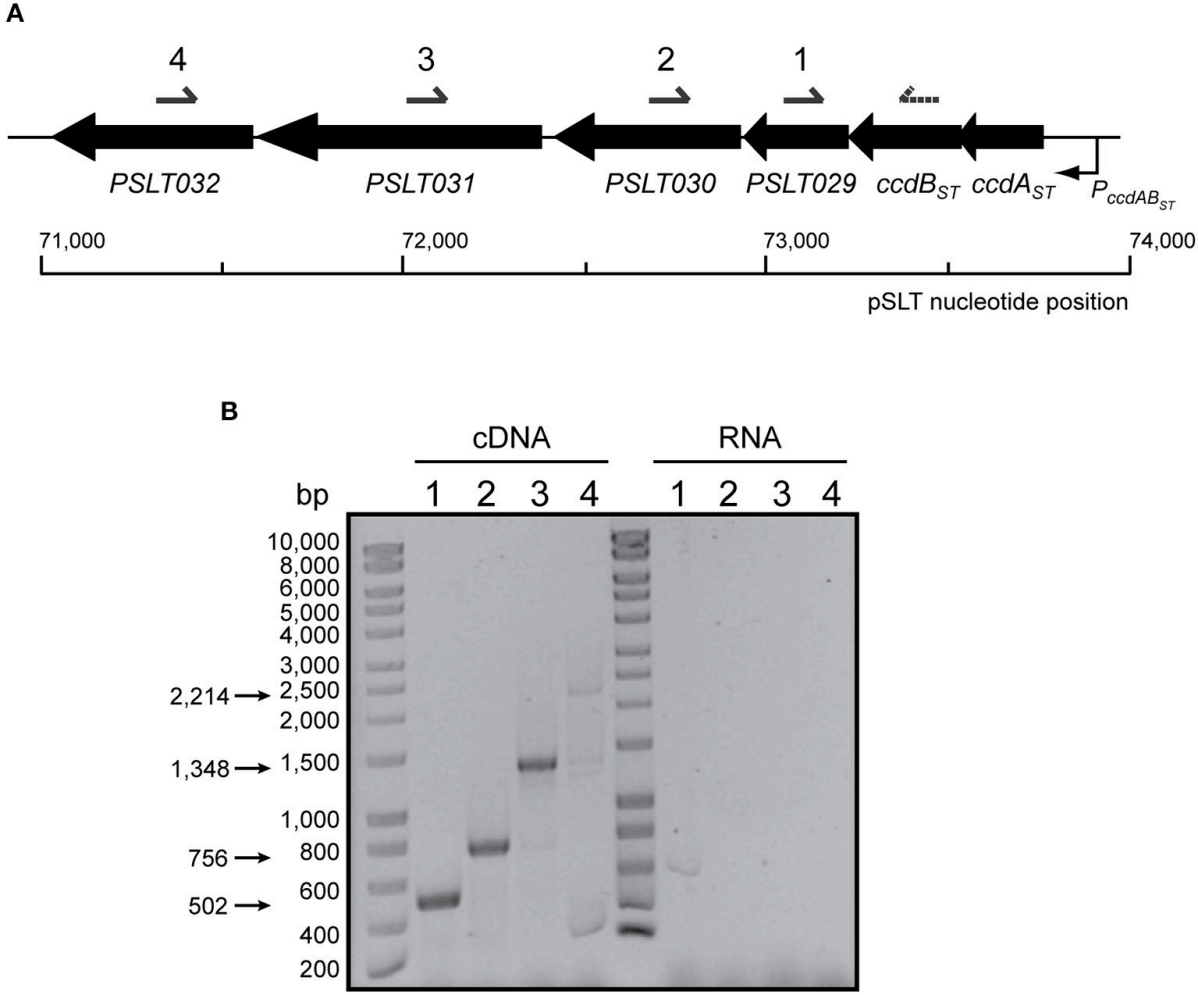
**Genetic organization and transcriptional analysis of the *ccdAB*_ST_ locus present in *S*. Typhimurium pSLT plasmid. (A)** Genetic organization of *ccdAB*_ST_ in *S*. Typhimurium pSLT. Black arrows represent the location and orientation of the genes (scaled). Under the scheme it is marked pSLT DNA sequence coordinates. Primers used for RT-PCR (number 4) and cDNA amplification (numbers 1–4) are denoted above. **(B)** Co-transcription analysis of *ccdAB*_ST_ and downstream genes. cDNA was synthesized using a primer annealing with *PSLT032* (named as 4). Primers named as 1, 2, 3, and 4 were used in combination to a primer annealing in 3′-end of *ccdB*_ST_ (dashed arrow) to PCR-amplify each region, and PCR products were resolved in a 0.8% agarose gel. DNA marker is shown on the left. The expected sizes of PCR-amplified DNA fragments are indicated with arrows on the left. All genes are co-transcribed with those encoding the *ccdAB*_ST_ TA system.

In the *E. coli* F plasmid, *ccdAB* maps upstream of the resolvase-encoding gene *resD*. However, analysis of the regions flanking *ccdAB*_ST_ TA system of pSLT showed that this locus could be genetically linked to four other downstream genes (Figure 5A). *ccdB*_ST_ gene is separated by only one single nucleotide from the downstream gene *SL1344_P1_0078* (*PSLT029*), which itself overlaps 4 bp with *SL1344_P1_0077* (*PSLT030*). The next downstream gene is *SL1344_P1_0076* (*PSLT031* or *rsdB*). *PSLT031* maps 33 bp downstream from the 3′-end of *SL1344_P1_0077* (*PSLT030*) and 8 bp upstream from the 5′-end of *SL1344_P1_0075* (*PSLT032*) (Figure 5A). These short intergenic regions led us hypothesize that the TA system *ccdAB*_ST_ of pSLT plasmid could be encoded within a six-gene polycistronic operon. RT-PCR assays confirmed a polycistronic operon encompassing from *ccdAB*_ST_ to *PSLT032* (Figure 5B).

### *ccdAB*_ST_ transcriptional regulation is important to control the polycistronic operon

**Figure 6:**
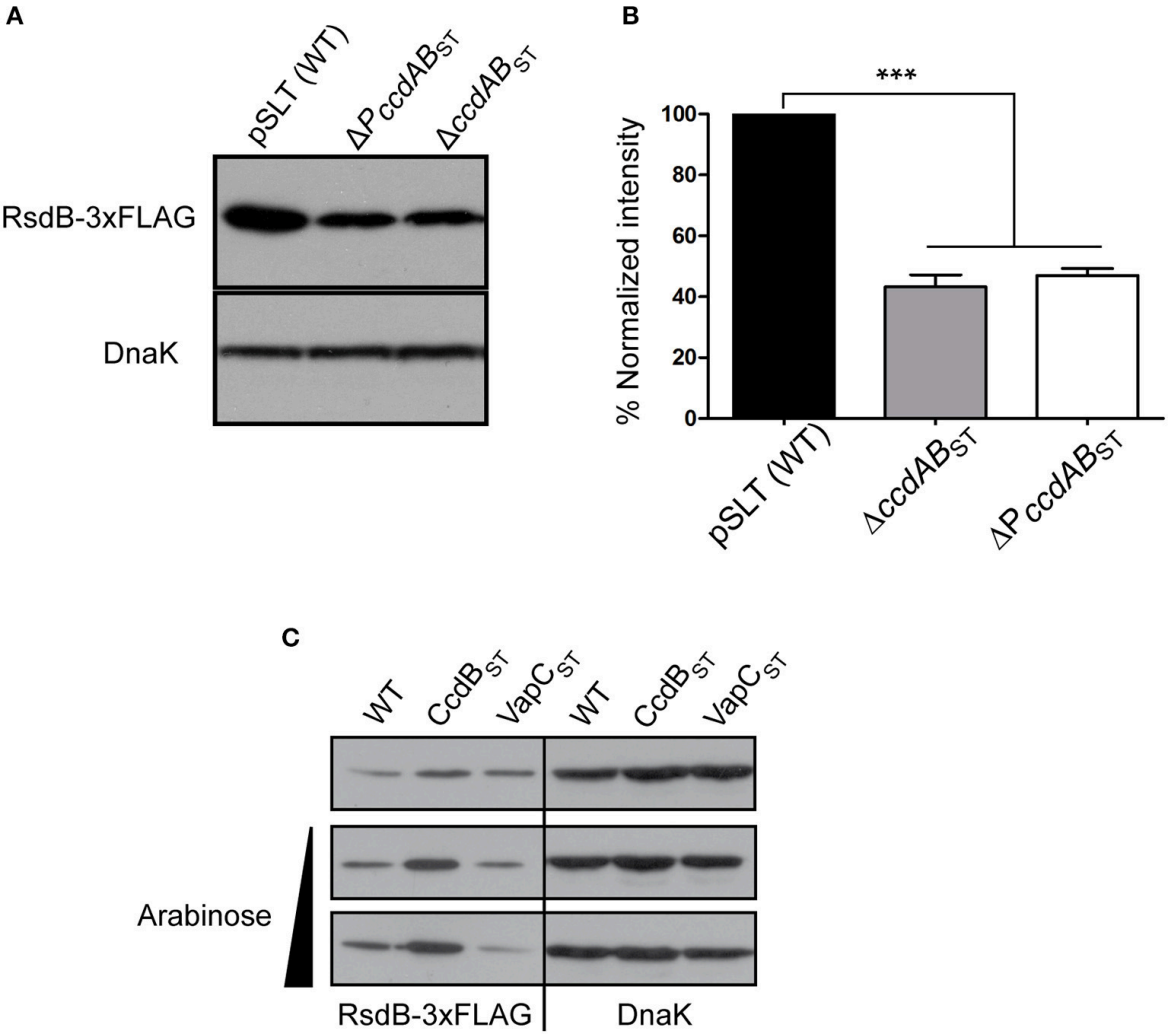
***ccdAB*_ST_ TA system influences the transcription of the polycistronic operon**. *rsdB* of pSLT plasmid was tagged with a 3xFLAG epitope in its 3′-end. **(A,B)** RsdB-3xFLAG levels were analyzed in pSLT plasmid lacking the *PccdAB*_ST_ promoter or the entire *ccdAB*_ST_ locus (including toxin, antitoxin and the promoter of the system). Bands intensity in **(A)** were quantified taking into account the number of cells loaded in the acrylamide gels and the band intensity of the loading control DnaK **(B)** Deletion of either the *ccdAB*_ST_ operon or the promoter of the system, *PccdAB*_ST_, reduced RsdB 2.3 and 2.1 fold, respectively. **(C)** Conditional cooperativity phenomenon was analyzed in a wild type pSLT carrying the tagged 3xFLAG *rsdB*. CcdB_ST_ or VapC_ST_ (negative control) proteins were supplement *in trans* from pCcdB and pVapC plasmids. Due to the conditional cooperativity phenomenom, overexpression of CcdB_ST_ results in derepression of *PccdAB*_ST_ and increased amount of RsdB. Quantification was carried out as described for A and B. Protein expression of CcdB_ST_ and VapC_ST_ was confirmed in SDS-acrylamide gels stained with Coomassie blue (Supplementary Figure 1). Histograms represent the quantification of at least four independent experiments. ^***^p < 0.001 by one-way ANOVA.

To further analyze the role of *ccdAB*_ST_ in the polycistronic operon transcriptional control we asked if placed at the beginning of the operon, CcdA_ST_-CcdB_ST_ TA complexes could modulate transcriptional expression of the operon in a TA system “classic” manner. *PSLT031* or *rsdB*, placed at the penultimate position of the polycistronic operon, is annotated as a putative resolvase that could be important in multimer resolution during pSLT plasmid replication (Krause and Guiney, 1991). Thus, we used *rsdB* as a reporter to monitor the operon transcriptional regulation exerted by *ccdAB*_ST_. We tagged the *rsdB* gene with a 3xFLAG epitope at the 3′-end, and measured its protein levels in strains carrying: (i) wild type pSLT, (ii) engineered pSLT lacking the whole *ccdAB*_ST_, and (iii) pSLT lacking the 300 bp containing the *ccdAB*_ST_ promoter. RsdB levels significantly decreased when the *ccdAB*_ST_ TA system was altered, thus indicating that *ccdAB*_ST_ acts as transcriptional repressor for the polycistronic operon (Figures 6A,B). As described above for the *ccdAB*_ST_ TA system, we tested if the polycistronic operon could also respond to conditional cooperativity. We expressed the non-toxic CcdB_ST_ variant and measured RsdB levels. Complementary, we used as a negative control the production of the unrelated toxin VapC_ST_. When CcdB_ST_ was provided *in trans* (Supplementary Figure 1), RsdB levels increased accordingly to conditional cooperativity (Figure 6C). Altogether, these data demonstrate that CcdAB_ST_ TA complexes influence the transcription of the polycistronic operon.

## Discussion

In this report we describe a novel method to measure plasmid stability in bacteria. This procedure is based on the use of an *aph*-*parE* cassette in which a rhamnose-inducible promoter controls synthesis of ParE toxin. When the *aph*-*parE* cassette is inserted in the plasmid of interest and rhamnose is present in the medium as the only carbon source, ParE is synthesized and plasmid-containing cells are selectively eliminated. This methodology allows direct selection of plasmid-free segregants in a reproducible and highly sensitive manner. As it has been described previously for many low-copy-number plasmids, the pSLT virulence plasmid of *S*. Typhimurium possesses at least three main mechanisms to ensure its stable maintenance in the cell:(i) a copy number control of replication mediated by *repB* and *repC* replicons; (ii) the *parAB* partition system; and (iii) the TA systems *ccdAB*_ST_ and *vapBC2*_ST_. In our study, we did not considered the influence of the conjugation machinery because although *S*. Typhimurium SV5015 pSLT is mobilizable, it is not self-transmissible (Ahmer et al., 1999). Using our novel stability assay, we reevaluated the contribution of ParAB and CcdAB_ST_ to pSLT plasmid stability as a proof of concept for the reliability of our methodology. In accordance with the literature, we show that the ParAB partition system stabilizes the pSLT plasmid very efficiently. Moreover, as described for other plasmids, the partition system appeared more important for pSLT stability than the *vapBC2*_ST_ or *ccdAB*_ST_ TA systems (Sia et al., 1995; Sengupta and Austin, 2011; Hernández-Arriaga et al., 2014). Several studies have demonstrated a moderately stabilizing effect of TA systems. Two examples are the *ccdAB* TA module of the fertility factor F (Ogura and Hiraga, 1983) and the *kis-kid* (also called *parD*) TA locus of the R1 plasmid (Bravo et al., 1987). These systems increase the stability of their host plasmids around 10-fold compared to mini-derivate plasmids (Hernández-Arriaga et al., 2014). However, there are exceptions to this rule. For instance, the *parDE* module of RK2 has a more important role in the stabilization of this plasmid than other TA systems (Roberts et al., 1994; Easter et al., 1997). Interestingly, the *mvpTA* TA system of the virulence plasmid pWR100 in *S. flexneri* is the principal contributor to plasmid stability, more than the partition system (Sayeed et al., 2005). This differs from the stability contribution of its ortholog in *S*. Typhimurium, *vapBC2*_ST_. Of note, MvpAT and VapBC2_ST_ show more than 96% amino acid sequence identity. However, it has also been described that diverse experimental variables, including temperature, growth media or the strain analyzed in the assay can alter plasmid stability (Easter et al., 1997; Sayeed et al., 2005). The toxin MvpT is a specific endonuclease that cleaves the initiator tRNA (Winther and Gerdes, 2011), and the *mvpTA* TA system has been shown to stabilize the virulence plasmid of *S. flexneri* by post-segregational killing (Sayeed et al., 2000). On the other hand, the plasmidic toxin VapC2_ST_ and its chromosomal paralog VapC_ST_ of *S*. Typhimurium conserve 82% amino acid sequence identity (Lobato-Márquez et al., 2015). Moreover, similar to MvpT toxin, the chromosomal VapC_ST_ toxin possesses tRNA endonuclease activity (Winther and Gerdes, 2011). These evidences imply that VapBC2_ST_ may mediate pSLT plasmid stability by post-segregational killing.

The other TA system of pSLT is *ccdAB*_ST_. In this work we demonstrate that this TA module shows classic characteristics of type II TA loci, such as autorepression and conditional cooperativity. Moreover, *ccdAB*_ST_ is highly conserved to its ortholog present in the F plasmid: 90 and 83% amino acid identity to CcdA and CcdB, respectively. One important amino acid substitution is the tryptophan 99 to arginine in CcdB_ST_ of pSLT, an indispensable residue for the toxic activity of CcdB (Bahassi et al., 1995). Using a pSLT plasmid derivate encoding a CcdB_ST_ (R99W) variant we demonstrate that CcdB_ST_ toxicity is not necessary for the contribution of this TA module to plasmid stability. Intriguingly, *ccdAB*_ST_ forms part of a polycistronic operon with four other downstream genes. Moreover, CcdA_ST_-CcdB_ST_ TA complexes contribute to the regulation of the expression of this operon. This result is surprising given that few exceptions escape the general rule of TA operons organization. These exceptions include TA modules with a third gene acting as the transcriptional repressor of the system (Zielenkiewicz and Ceglowski, 2005; Hallez et al., 2010) and a single case in which a chaperone, co-transcribed with a TA operon, facilitates the folding of the antitoxin and, therefore, its activity (Bordes et al., 2011). Although, RsdB levels decreased upon deletion of either the promoter of *ccdAB*_ST_ TA module or the whole TA locus, we still detected RsdB by western blot. These results indicate that *PccdAB*_ST_ does control the transcription of the operon but it may exist at least another additional promoter regulating the operon.

Future work should address how the unprecedented TA genomic organization of this novel polycistronic operon including *ccdAB*_*ST*_ and its transcriptional regulation influence pSLT stability. pSLT is evolutionary related to F plasmid, yet in F *ccdAB* does not constitute such a polycistronic operon. The study of this particular TA system could shed light on the evolution and adaptation of TA modules to its bacterial host.

## Materials and methods

### Bacterial strains, plasmids and growth conditions

*S. enterica* serovar Typhimurium SV5015 (a SL1344 His^+^ derivate strain Mariscotti and García-del Portillo, 2009) was used as parental strain (*S*. Typhimurium SL1344 accession number: NC_016810.1). All strains and plasmids used in this study are listed in Supplementary Table 1. Bacteria were grown at 37°C with shaking at 150 rpm in Luria-Bertani (LB) medium. When necessary antibiotics were added at the following concentrations: kanamycin, 50 μg/ml; ampicillin, 50 μg/ml; cloramphenicol, 20 μg/ml.

A transcriptional fusion *PccdAB*_ST_-*lacZ* was designed to measure the transcriptional activity of *PccdAB*_ST_ promoter. A 300 bp DNA sequence upstream of *ccdA*_ST_ containing the promoter of *ccdAB*_ST_ (Tam and Kline, 1989; Madl et al., 2006) was PCR-amplified, digested with EcoRI-KpnI and ligated with the large EcoRI-KpnI fragment of plasmid pMP220 (Spaink et al., 1987). The resulting plasmid was confirmed by DNA sequencing.

### Construction of *S*. Typhimurium mutants

Oligonucleotide primers used in these procedures are listed in Supplementary Table 2. For disruption of pSLT plasmid maintenance modules, the deletion method described by Maisonneuve et al., was used (Maisonneuve et al., 2011). The strain used as control on stability assays was design inserting an *aph-parE* cassette in the *spvA* gene of pSLT. Disruption of this gene does not alter pSLT stability (Ahmer et al., 1999; García-Quintanilla et al., 2006).

A similar protocol to that involving generation of deletion mutants was used to introduce the amino acid substitution R99W in CcdB_ST_. Briefly, the *aph-parE* module was first introduced in *ccdB*_ST_ gene. Then the cassette was cleaned up with a PCR-amplified DNA fragment bearing the nucleotide change C–T in the position 73,232 of pSLT plasmid corresponding to the first nucleotide of the arginine 99 (R99) codon.

Construction of *S*. Typhimurium recombinant strain expressing tagged RsdB-3xFLAG was carried out as previously described (Uzzau et al., 2001). 3x-FLAG tagging was performed at the 3′-end of the *PSLT031* gene.

All mutants were verified and confirmed by PCR.

### Plasmid stability assays

Before starting stability assays, bacteria were grown in LB containing 50 μg/ml kanamycin. For plasmid stability assays all bacterial strains were grown in 10 ml LB medium (10:1 flask:medium volume ratio) without selection pressure for 16 h (~10 generations) at 37°C and 150 rpm. We did not observe alterations in the growth rate of the pSLT plasmid derivates lacking *parAB, ccdAB*_ST_ or *vapBC2*_ST_ compared to pSLT wild type plasmid. Aliquots of 1 ml of the culture were collected into 1.5 ml eppendorf tubes and bacteria were pelleted in a MiniSpin® Eppendorf centrifuge 1 min at 12,000 rpm at room temperature. Supernatants were discarded and bacterial pellets were washed twice with phosphate buffered saline (PBS) pH 7.4. This ensures proper elimination of LB medium traces that otherwise could interfere with the growth in M9-rhamnose plates. Serial dilutions were done in PBS pH 7.4 and 100 μl of the appropriate aliquots plated onto LB- or M9-rhamnose-agar plates. Typically a 1:10^7^ dilution was used to quantify total bacterial population in LB-agar plates, and dilutions in the range 1:1–10^3^ were used to determine the number of segregants in M9-rhamnose-agar plates. Plates were incubated for 24 h (LB-agar) or 48–72 h (M9-rhamnose-agar) at 37°C before counting of the colony forming units. Colony forming units grown in M9-rhamnose-agar were tested for their kanamycin resistance on antibiotic-containing LB plates. This is a sensitive assay that effectively eliminates plasmid-containing cells, thus allowing a direct selection of plasmid-free segregants.

### β-galactosidase activity measurements

Bacteria containing the plasmid with transcriptional fusion *PccdAB*-*lacZ* were grown to an optical density (OD)_600_ of 0.6 at 37°C and 150 rpm in LB. Then, β-galactosidase activity was measured as previously described (Miller, 1972).

For the conditional cooperativity experiments, bacteria containing pCcdB or pVapC plasmids (Lobato-Márquez et al., 2015) were grown in LB to an OD_600_ of 0.3 at 37°C and 150 rpm in the presence of 50 μg/ml kanamycin. Inactive CcdB_ST_ or VapC_ST_ toxins were synthesized upon induction with 0.3 % (w/v) L-arabinose. β-galactosidase activity was assessed as in the rest of strains after 1 h of induction. The chromosomally-encoded *S*. Typhimurium VapC_ST_ was used as a control to discard unspecific effects of protein expression in β-galactosidase measurements.

### Reverse transcriptase PCR (RT-PCR)

To determine the presence of a polycistronic operon controlled by *ccdAB*_ST_ total RNA was extracted from wild type *S*. Typhimurium SV5015 (Mariscotti and García-del Portillo, 2009) grown in LB at 37°C until OD_600_ ~ 0.3. Volume corresponding to 1 absorbance unit at OD_600_ was lysed in 100 μl lysis buffer (lysozime 50 mg/ml, 0.3% SDS). Cells extracts were processed using *RNeasy minit kit* (#74104, Quiagen). cDNA was constructed employing ThermoScript RT-PCR system (#11146-016, Invitrogen), using 600 ng of total RNA as template, a t_m_ of 60°C and 0.6 μM of a oligonucleotide annealing with the 3′-end of *PSLT032* (Supplementary Table 2). cDNA was amplified by PCR (Pfu DNA polymerase, #M774B, Promega) using 0.5 μM of primers annealing with *ccdB*_ST_, *SL1344_P1_0078* (*PSLT029*), *SL1344_P1_0077* (*PSLT030*), *rsdB*, and *SL1344_P1_0075* (*PSLT032*) (Supplementary Table 2). PCR amplification was carried out in duplicate using cDNA and RNA as a negative control. PCR products were visualized in 0.8% (w/v) agarose gels stained with ethidium bromide.

### Detection of RsdB levels by western blotting and protein levels quantification

Bacterial cultures were grown 16 h at 37°C and 150 rpm. Same amount of bacterial cells were collected (volumes were adjusted based on OD_600_), centrifuged (1 min at 12,000 rpm) and re-suspend in Laemmli buffer (Laemmli, 1970). Bacterial protein extracts were resolved in SDS-PAGE using 15% polyacrylamide gels and processed for Western blot assays. Levels of the *S*. Typhimurium DnaK protein were used as loading control. RsdB or DnaK detection were performed using anti-FLAG antibody (#F3165, Sigma-Aldrich) 1:2000 (2 h) or anti-DNAK 1:10,000 (1 h), respectively, disolved in TBS-Tween buffer (137 mM NaCl, 0.1% m/v Tween 20 and 20 mM Tris-HCl pH 7.5) containing 3% non-fat milk. RsdB expression levels were calculated by western blotting experiments using extracts prepared from at least four independent experiments and pSLT plasmid variants expressing 3xFLAG-tagged RsdB. Mean data were taken as the relative expression levels of the proteins. Band densitometry was determined using Quantity One v.4.6.3 software (Bio-Rad, Berkeley, CA) as previously described (Molina-García and Giraldo, 2014; López-Villarejo et al., 2015).

### Statistical analyses

Statistical significance was analyzed with GraphPad Prism v7 software (GraphPad Inc., La Jolla, CA) using one-way analysis of variance (ANOVA) with Dunnett's multiple comparison post-test for Figures 2, 4, 6B. In the comparison test used for Figure 3B a Student's *T*-test analysis was used. A *P* ≤ 0.05 was considered significant. Data are presented as mean ± standard deviation of the mean (SEM).

## Author contributions

DL and RD: Conceived and designed the experiments; DL, LM, and IM: Performed the experiments; DL, LM, IM, FG, and RD: Analyzed the data; DL: Wrote the paper.

### Conflict of interest statement

The authors declare that the research was conducted in the absence of any commercial or financial relationships that could be construed as a potential conflict of interest. The reviewer GDS declared a shared affiliation, though no other collaboration, with several of the authors IM, RD to the handling Editor, who ensured that the process nevertheless met the standards of a fair and objective review.

## Supplementary material

Supplementary Figure 1 **Control assays showing proper protein synthesis of CcdB_ST_ and VapC_ST_ in the experiments involving conditional cooperativity regulation of *rsdB* in Figure 6C (main text)**. Equal amounts of total protein extracts were loaded in each lane. Bacteria were grown in LB medium to OD_600_ of 0.3, time at which CcdB_ST_ or VapC_ST_ expression was induced with 0.3% arabinose.

Supplementary Table 1 **Bacterial strains and plasmids used in this study**.

Supplementary Table 2 **Oligonucleotides used in this study**.

## References

[B1] Ahmer B. M. Tran M. Heffron F. (1999). The virulence plasmid of *Salmonella* Typhimurium is self-transmissible. J. Bacteriol. 181, 1364–1368. 9973370 10.1128/jb.181.4.1364-1368.1999 PMC93521

[B2] Austin S. Abeles A. (1983). Partition of unit-copy miniplasmids to daughter cells. I. P1 and F miniplasmids contain discrete, interchangeable sequences sufficient to promote equipartition. J. Mol. Biol. 169, 353–372. 10.1016/S0022-2836(83)80055-2 6312056

[B3] Bahassi E. M. Salmon M. A. Van Melderen L. Bernard P. Couturier M. (1995). F plasmid CcdB killer protein: *ccdB* gene mutants coding for non-cytotoxic proteins which retain their regulatory functions. Mol. Microbiol. 15, 1031–1037. 10.1111/j.1365-2958.1995.tb02278.x 7623659

[B4] Bäumler A. J. Tsolis R. M. Ficht T. A. Adams L. G. (1998). Evolution of host adaptation in *Salmonella enterica*. Infect. Immun. 66, 4579–4587. 9746553 10.1128/iai.66.10.4579-4587.1998 PMC108564

[B5] Bochner B. R. Huang H. C. Schieven G. L. Ames B. N. (1980). Positive selection for loss of tetracycline resistance. J. Bacteriol. 143, 926–933. 6259126 10.1128/jb.143.2.926-933.1980 PMC294396

[B6] Bordes P. Cirinesi A. M. Ummels R. Sala A. Sakr S. Bitter W.. (2011). SecB-like chaperone controls a toxin-antitoxin stress-responsive system in *Mycobacterium tuberculosis*. Proc. Natl. Acad. Sci. U.S.A. 108, 8438–8443. 10.1073/pnas.1101189108 21536872 PMC3100995

[B7] Bravo A. de Torrontegui G. Díaz R. (1987). Identification of components of a new stability system of plasmid R1, ParD, that is close to the origin of replication of this plasmid. Mol. Gen. Genet. 210, 101–110. 10.1007/BF00337764 3323833

[B8] Chan W. T. Espinosa M. Yeo C. C. (2016). Keeping the wolves at Bay: antitoxins of prokaryotic type ii toxin-antitoxin systems. Front. Mol. Biosci. 3:9. 10.3389/fmolb.2016.00009 27047942 PMC4803016

[B9] Dao-Thi M. H. Van Melderen L. De Genst E. Afif H. Buts L. Wyns L.. (2005). Molecular basis of gyrase poisoning by the addiction toxin CcdB. J. Mol. Biol. 348, 1091–1102. 10.1016/j.jmb.2005.03.049 15854646

[B10] del Solar G. H. Puyet A. Espinosa M. (1987). Initiation signals for the conversion of single stranded to double stranded DNA forms in the streptococcal plasmid pLS1. Nucleic Acids Res. 15, 5561–5580. 10.1093/nar/15.14.5561 3039461 PMC306007

[B11] Easter C. L. Sobecky P. A. Helinski D. R. (1997). Contribution of different segments of the *par* region to stable maintenance of the broad-host-range plasmid RK2. J. Bacteriol. 179, 6472–6479. 9335298 10.1128/jb.179.20.6472-6479.1997 PMC179565

[B12] Ebersbach G. Gerdes K. (2005). Plasmid segregation mechanisms. Annu. Rev. Genet. 39, 453–479. 10.1146/annurev.genet.38.072902.091252 16285868

[B13] García-Quintanilla M. Prieto A. I. Barnes L. Ramos-Morales F. Casadesús J. (2006). Bile-induced curing of the virulence plasmid in *Salmonella enterica* serovar Typhimurium. J. Bacteriol. 188, 7963–7965. 10.1128/JB.00995-06 16963576 PMC1636308

[B14] Gerdes K. Larsen J. E. Molin S. (1985). Stable inheritance of plasmid R1 requires two different loci. J. Bacteriol. 161, 292–298. 2981804 10.1128/jb.161.1.292-298.1985 PMC214870

[B15] Gerdes K. Rasmussen P. B. Molin S. (1986). Unique type of plasmid maintenance function: postsegregational killing of plasmid-free cells. Proc. Natl. Acad. Sci. U.S.A. 83, 3116–3120. 10.1073/pnas.83.10.3116 3517851 PMC323463

[B16] Grimont P. Weill F. (2007). Antigenic Formulae of the Salmonella Serovars, 9th Edn. Paris: World Health Organization Collaborating Center for Reference and Research on Salmonella. Pasteur Institute.

[B17] Gulig P. A. Danbara H. Guiney D. G. Lax A. J. Norel F. Rhen M. (1993). Molecular analysis of *spv* virulence genes of the *Salmonella* virulence plasmids. Mol. Microbiol. 7, 825–830. 10.1111/j.1365-2958.1993.tb01172.x 8483415

[B18] Hallez R. Geeraerts D. Sterckx Y. Mine N. Loris R. Van Melderen L. (2010). New toxins homologous to ParE belonging to three-component toxin-antitoxin systems in *Escherichia coli* O157:H7. Mol. Microbiol. 76, 719–732. 10.1111/j.1365-2958.2010.07129.x 20345661

[B19] Hernández-Arriaga A. M. Chan W. T. Espinosa M. Díaz-Orejas R. (2014). Conditional activation of toxin-antitoxin systems: postsegregational killing and beyond. Microbiol. Spectr. 2:PLAS-0009-2013. 10.1128/microbiolspec.PLAS-0009-2013 26104348

[B20] Jackson R. W. Vinatzer B. Arnold D. L. Dorus S. Murillo J. (2011). The influence of the accessory genome on bacterial pathogen evolution. Mob. Genet. Elem. 1, 55–65. 10.4161/mge.1.1.16432 22016845 PMC3190274

[B21] Jiang Y. Pogliano J. Helinski D. R. Konieczny I. (2002). ParE toxin encoded by the broad-host-range plasmid RK2 is an inhibitor of *Escherichia coli* gyrase. Mol. Microbiol. 44, 971–979. 10.1046/j.1365-2958.2002.02921.x 12010492

[B22] Jones G. W. Rabert D. K. Svinarich D. M. Whitfield H. J. (1982). Association of adhesive, invasive, and virulent phenotypes of *Salmonella* Typhimurium with autonomous 60-megadalton plasmids. Infect. Immun. 38, 476–486. 6128304 10.1128/iai.38.2.476-486.1982 PMC347764

[B23] Kline B. C. (1985). A review of mini-F plasmid maintenance. Plasmid 14, 1–16. 10.1016/0147-619X(85)90027-7 3898165

[B24] Krause M. Guiney D. G. (1991). Identification of a multimer resolution system involved in stabilization of the *Salmonella* Dublin virulence plasmid pSDL2. J. Bacteriol. 173, 5754–5762. 1653217 10.1128/jb.173.18.5754-5762.1991 PMC208307

[B25] Laemmli U. K. (1970). Cleavage of structural proteins during the assembly of the head of bacteriophage T4. Nature 227, 680–685. 10.1038/227680a0 5432063

[B26] Li X. T. Thomason L. C. Sawitzke J. A. Costantino N. Court D. L. (2013). Positive and negative selection using the *tetA-sacB* cassette: recombineering and P1 transduction in *Escherichia coli*. Nucleic Acids Res. 41, e204. 10.1093/nar/gkt1075 24203710 PMC3905872

[B27] Lobato-Márquez D. Díaz-Orejas R. García-del Portillo F. (2016). Toxin-antitoxins and bacterial virulence. FEMS Microbiol. Rev. 40, 592–609. 10.1093/femsre/fuw022 27476076

[B28] Lobato-Márquez D. Moreno-Córdoba I. Figueroa V. Díaz-Orejas R. García-del Portillo F. (2015). Distinct type I and type II toxin-antitoxin modules control *Salmonella* lifestyle inside eukaryotic cells. Sci. Rep. 5:9374. 10.1038/srep09374 25792384 PMC4366850

[B29] López-Villarejo J. Lobato-Márquez D. Díaz-Orejas R. (2015). Coupling between the basic replicon and the Kis-Kid maintenance system of plasmid R1: modulation by Kis antitoxin levels and involvement in control of plasmid replication. Toxins (Basel). 7, 478–492. 10.3390/toxins7020478 25664511 PMC4344636

[B30] Madl T. Van Melderen L. Mine N. Respondek M. Oberer M. Keller W.. (2006). Structural basis for nucleic acid and toxin recognition of the bacterial antitoxin CcdA. J. Mol. Biol. 364, 170–185. 10.1016/j.jmb.2006.08.082 17007877

[B31] Maisonneuve E. Shakespeare L. J. Jørgensen M. G. Gerdes K. (2011). Bacterial persistence by RNA endonucleases. Proc. Natl. Acad. Sci. U.S.A. 108, 13206–13211. 10.1073/pnas.1100186108 21788497 PMC3156201

[B32] Makino S. Sasakawa C. Yoshikawa M. (1988). Genetic relatedness of the basic replicon of the virulence plasmid in *shigellae* and enteroinvasive *Escherichia coli*. Microb. Pathog. 5, 267–274. 10.1016/0882-4010(88)90099-X 3070262

[B33] Maloy S. R. Nunn W. D. (1981). Selection for loss of tetracycline resistance by *Escherichia coli*. J. Bacteriol. 145, 1110–1111. 7007341 10.1128/jb.145.2.1110-1111.1981 PMC217228

[B34] Mariscotti J. F. García-del Portillo F. (2009). Genome expression analyses revealing the modulation of the *Salmonella* Rcs regulon by the attenuator IgaA. J. Bacteriol. 191, 1855–1867. 10.1128/JB.01604-08 19124574 PMC2648367

[B35] Miller J. H. (1972). Experiments in Molecular Genetics. New York, NY: Cold Spring Harbor Laboratory.

[B36] Million-Weaver S. Camps M. (2014). Mechanisms of plasmid segregation: have multicopy plasmids been overlooked? Plasmid 75, 27–36. 10.1016/j.plasmid.2014.07.002 25107339 PMC4163524

[B37] Molina-García L. Giraldo R. (2014). Aggregation interplay between variants of the RepA-WH1 prionoid in *Escherichia coli*. J. Bacteriol. 196, 2536–2542. 10.1128/JB.01527-14 24794561 PMC4097580

[B38] Ogura T. Hiraga S. (1983). Mini-F plasmid genes that couple host cell division to plasmid proliferation. Proc. Natl. Acad. Sci. U.S.A. 80, 4784–4788. 10.1073/pnas.80.15.4784 6308648 PMC384129

[B39] Overgaard M. Borch J. Jørgensen M. G. Gerdes K. (2008). Messenger RNA interferase RelE controls *relBE* transcription by conditional cooperativity. Mol. Microbiol. 69, 841–857. 10.1111/j.1365-2958.2008.06313.x 18532983

[B40] Reyrat J. M. Pelicic V. Gicquel B. Rappuoli R. (1998). Counterselectable markers: untapped tools for bacterial genetics and pathogenesis. Infect. Immun. 66, 4011–4017. 9712740 10.1128/iai.66.9.4011-4017.1998 PMC108478

[B41] Rivera-Chávez F. Bäumler A. J. (2015). The pyromaniac inside you: *Salmonella* metabolism in the host gut. Annu. Rev. Microbiol. 69, 31–48. 10.1146/annurev-micro-091014-104108 26002180

[B42] Roberts R. C. Ström A. R. Helinski D. R. (1994). The *parDE* operon of the broad-host-range plasmid RK2 specifies growth inhibition associated with plasmid loss. J. Mol. Biol. 237, 35–51. 10.1006/jmbi.1994.1207 8133518

[B43] Rotger R. Casadesús J. (1999). The virulence plasmids of *Salmonella*. Int. Microbiol. 2, 177–184. 10943411

[B44] Salmon M. A. Van Melderen L. Bernard P. Couturier M. (1994). The antidote and autoregulatory functions of the F plasmid CcdA protein: a genetic and biochemical survey. Mol. Gen. Genet. 244, 530–538. 10.1007/BF00583904 8078480

[B45] Sasakawa C. Kamata K. Sakai T. Murayama S. Y. Makino S. Yoshikawa M. (1986). Molecular alteration of the 140-megadalton plasmid associated with loss of virulence and Congo red binding activity in *Shigella flexneri*. Infect. Immun. 51, 470–475. 3002985 10.1128/iai.51.2.470-475.1986 PMC262355

[B46] Sayeed S. Brendler T. Davis M. Reaves L. Austin S. (2005). Surprising dependence on postsegregational killing of host cells for maintenance of the large virulence plasmid of *Shigella flexneri*. J. Bacteriol. 187, 2768–2773. 10.1128/JB.187.8.2768-2773.2005 15805523 PMC1070380

[B47] Sayeed S. Reaves L. Radnedge L. Austin S. (2000). The stability region of the large virulence plasmid of *Shigella flexneri* encodes an efficient postsegregational killing system. J. Bacteriol. 182, 2416–2421. 10.1128/JB.182.9.2416-2421.2000 10762240 PMC111302

[B48] Sengupta M. Austin S. (2011). Prevalence and significance of plasmid maintenance functions in the virulence plasmids of pathogenic bacteria. Infect. Immun. 79, 2502–2509. 10.1128/IAI.00127-11 21555398 PMC3191983

[B49] Sia E. A. Roberts R. C. Easter C. Helinski D. R. Figurski D. H. (1995). Different relative importances of the *par* operons and the effect of conjugal transfer on the maintenance of intact promiscuous plasmid RK2. J. Bacteriol. 177, 2789–2797. 7751288 10.1128/jb.177.10.2789-2797.1995 PMC176950

[B50] Spaink H. P. Okker R. J. Wijffelman C. A. Pees E. Lugtenberg B. J. (1987). Promoters in the nodulation region of the *Rhizobium leguminosarum* Sym plasmid pRL1JI. Plant Mol. Biol. 9, 27–39. 10.1007/BF00017984 24276795

[B51] Tam J. E. Kline B. C. (1989). Control of the *ccd* operon in plasmid F. J. Bacteriol. 171, 2353–2360. 2651399 10.1128/jb.171.5.2353-2360.1989 PMC209908

[B52] Tindall B. J. Grimont P. A. Garrity G. M. Euzeby J. P. (2005). Nomenclature and taxonomy of the genus *Salmonella*. Int. J. Syst. Evol. Microbiol. 55(Pt 1), 521–524. 10.1099/ijs.0.63580-0 15653930

[B53] Tinge S. A. Curtiss R. III. (1990a). Conservation of *Salmonella* Typhimurium virulence plasmid maintenance regions among *Salmonella* serovars as a basis for plasmid curing. Infect. Immun. 58, 3084–3092. 2167294 10.1128/iai.58.9.3084-3092.1990 PMC313615

[B54] Tinge S. A. Curtiss R. III. (1990b). Isolation of the replication and partitioning regions of the *Salmonella* Typhimurium virulence plasmid and stabilization of heterologous replicons. J. Bacteriol. 172, 5266–5277. 2203747 10.1128/jb.172.9.5266-5277.1990 PMC213189

[B55] Uzzau S. Figueroa-Bossi N. Rubino S. Bossi L. (2001). Epitope tagging of chromosomal genes in *Salmonella*. Proc. Natl. Acad. Sci. U.S.A. 98, 15264–15269. 10.1073/pnas.261348198 11742086 PMC65018

[B56] Van Melderen L. Bernard P. Couturier M. (1994). Lon-dependent proteolysis of CcdA is the key control for activation of CcdB in plasmid-free segregant bacteria. Mol. Microbiol. 11, 1151–1157. 10.1111/j.1365-2958.1994.tb00391.x 8022284

[B57] Winther K. S. Gerdes K. (2011). Enteric virulence associated protein VapC inhibits translation by cleavage of initiator tRNA. Proc. Natl. Acad. Sci. U.S.A. 108, 7403–7407. 10.1073/pnas.1019587108 21502523 PMC3088637

[B58] Yamaguchi Y. Inouye M. (2011). Regulation of growth and death in *Escherichia coli* by toxin-antitoxin systems. Nat. Rev. Microbiol. 9, 779–790. 10.1038/nrmicro2651 21927020

[B59] Zielenkiewicz U. Ceglowski P. (2005). The toxin-antitoxin system of the streptococcal plasmid pSM19035. J. Bacteriol. 187, 6094–6105. 10.1128/JB.187.17.6094-6105.2005 16109951 PMC1196172

