# Supplementary figures and images for: Stabilization of the Virulence Plasmid pSLT of Salmonella Typhimurium by Three Maintenance Systems and Its Evaluation by Using a New Stability Test
Source: Front Mol Biosci. 2016 Oct 17;3:66. doi: 10.3389/fmolb.2016.00066 (PMC5065971; doi:10.3389/fmolb.2016.00066)

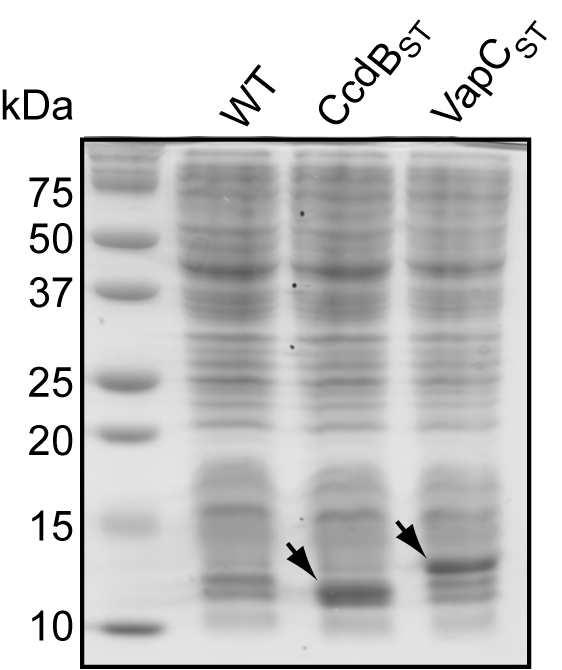

Supplement: Supplementary Figure 1 — Control assays showing proper protein synthesis of CcdBST and VapCST in the experiments involving conditional cooperativity regulation of rsdB in Figure 6C (main text). Equal amounts of total protein extracts were loaded in each lane. Bacteria were grown in LB medium to OD600 of 0.3, time at which CcdBST or VapCST expression was induced with 0.3% arabinose. [file Image1.TIF]
