# Supplementary material for: Stabilization of the Virulence Plasmid pSLT of Salmonella Typhimurium by Three Maintenance Systems and Its Evaluation by Using a New Stability Test
Source: Front Mol Biosci. 2016 Oct 17;3:66. doi: 10.3389/fmolb.2016.00066 (PMC5065971; doi:10.3389/fmolb.2016.00066)
Supplement: Supplementary Table 1 — Bacterial strains and plasmids used in this study. [file Table1.PDF]

Supplementary Table 1. Bacterial strains and plasmids used in this study.

**Bacterial species /**	**Strain**	**Relevant genotype**	**Reference**
*S.*Typhimurium	SV5015	SL1344 His^{+}	(Mariscotti and Garcia-del Portillo, 2009)
	SV5015-01	*ccdAB*$_{ST}$::*aph-parE*	This work
	SV5015-02	*ccdB*$_{ST}$::*aph-parE*	This work
	SV5015-03	P*ccdAB*$_{ST}$::*aph-parE*	This work
	SV5015-04	*vapBC2*$_{ST}$::*aph-parE*	This work
	SV5015-05	*parAB*::*aph-parE*	This work
	SV5015-07	Δ*ccdAB*$_{ST}$	This work
	SV5015-08	ΔP*ccdAB*$_{ST}$	This work
	SV5015-09	*rsdB*-3xFLAG	This work
	SV5015-10	Δ*ccdAB*$_{ST}$, *rsdB*-3xFLAG	This work
	SV5015-11	ΔP*ccdAB*$_{ST}$, *rsdB*-3xFLAG	This work
	SV3081	pSLT^{-}	(Torreblanca et al., 1999)
Plasmids	pCcdB	pBR322 derivate containing *ccdB*$_{ST}$, *P_{BAD}*, *aph*	(Lobato-Márquez et al., 2015)
	pVapC	pBR322 derivate containing *ccdB*$_{ST}$, *P_{BAD}*, *aph*	(Lobato-Márquez et al., 2015)
	pKD267	*aph-parE* cassette	(Maisonneuve et al., 2011)
	pKD46	*λred* recombinase, *P_{BAD}*, *amp*^{R}	(Datsenko and Wanner, 2000)
	pSUB11	*kanamycin-3xFLAG* cassette	(Uzzau et al., 2001)
	pCP20	*bla,* thermal induction of flipase synthesis, *amp*^{R}	(Cherepanov and Wackernagel, 1995)
	pMP220	Promoterless *lacZ, tet*	(Spaink et al., 1987)
	pPccdAB-lacZ	*PccdAB-lacZ* transcriptional fusion	This work

Supplementary references

Cherepanov, P.P., and Wackernagel, W. (1995). Gene disruption in *Escherichia coli*: TcR and KmR cassettes with the option of Flp-catalyzed excision of the antibiotic-resistance determinant. *Gene* 158(1)**,** 9-14.

Datsenko, K.A., and Wanner, B.L. (2000). One-step inactivation of chromosomal genes in *Escherichia coli* K-12 using PCR products. *Proc Natl Acad Sci USA* 97(12)**,** 6640-6645.

Lobato-Márquez, D., Moreno-Córdoba, I., Figueroa, V., Díaz-Orejas, R., and García-del Portillo, F. (2015). Distinct type I and type II toxin-antitoxin modules control *Salmonella* lifestyle inside eukaryotic cells. *Sci Rep* 5**,** 9374.

Maisonneuve, E., Shakespeare, L.J., Jorgensen, M.G., and Gerdes, K. (2011). Bacterial persistence by RNA endonucleases. *Proc Natl Acad Sci USA* 108(32)**,** 13206-13211.

Mariscotti, J.F., and García-del Portillo, F. (2009). Genome expression analyses revealing the modulation of the *Salmonella* Rcs regulon by the attenuator IgaA. *J Bacteriol* 191(6)**,** 1855-1867.

Spaink, H.P., Okker, R.J., Wijffelman, C.A., Pees, E., and Lugtenberg, B.J. (1987). Promoters in the nodulation region of the *Rhizobium leguminosarum* Sym plasmid pRL1JI. *Plant Mol Biol* 9(1)**,** 27-39.

Torreblanca, J., Marques, S., and Casadesus, J. (1999). Synthesis of FinP RNA by plasmids F and pSLT is regulated by DNA adenine methylation. *Genetics* 152(1)**,** 31-45.

Uzzau, S., Figueroa-Bossi, N., Rubino, S., and Bossi, L. (2001). Epitope tagging of chromosomal genes in *Salmonella*. *Proc Natl Acad Sci USA* 98(26)**,** 15264-15269.
