# Supplementary material for: Stabilization of the Virulence Plasmid pSLT of Salmonella Typhimurium by Three Maintenance Systems and Its Evaluation by Using a New Stability Test
Source: Front Mol Biosci. 2016 Oct 17;3:66. doi: 10.3389/fmolb.2016.00066 (PMC5065971; doi:10.3389/fmolb.2016.00066)
Supplement: Supplementary Table 2 — Oligonucleotides used in this study. [file Table2.PDF]

Supplementary Table 2. Oligonucleotides used in this study.

Name	Primer sequence (5′– 3′)	Used for
Fw-Del-ccdAB	TGACGGTGGCCGGCACACTGGTCATATCCGTGGTCAGCAGGCGGTAAGGCTCTCTACGCCGGACGCATCGTG	Amplification *aph-parE* cassette from pKD267 to delete *ccdAB*$_{ST}$
Rv-Del-ccdAB	TAAGAGATATGTAGATAATGGCGGCGAACAGTTAACTCAGTCACTATAAGACTGATCAGTGATAAGCTGTC	Amplification *aph-parE* cassette from pKD267 to delete *ccdAB*$_{ST}$
CcdAB-5.1	CCTGTGCAAACTGTTCTGTCGTCTC	Amplification *ccdAB*$_{ST}$ upstream region
CcdAB-3.1	GTTAACTCAGTCACTATAAGGCCTTACCGCCTGCTGACCAC	Amplification *ccdAB*$_{ST}$ upstream region
CcdAB-5.2	CGGGTCAGCAGGCGGTAAGGCCTTATAGTGACTGAGTTAACTG	Amplification *ccdAB*$_{ST}$ downstream region
CcdAB-3.2	GGACGACGGATGAAATATAAC	Amplification *ccdAB*$_{ST}$ downstream region
Fw-Del-ccdB	ATCAGGTTAATGGCGTTTTTGATATCGTTCTCCCGGAGGCTGAGATCGGCTCTCTACGCCGGACGCATCGTG	Amplification *aph-parE* cassette from pKD267 to delete *ccdB*$_{ST}$
Rv-Del-ccdB	ATGCAGTTTAAGGTTTACACCTGTAAAAGGGAGAGTCGCTACCGGCTGTTACTGATCAGTGATAAGCTGTC	Amplification *aph-parE* cassette from pKD267 to delete *ccdB*$_{ST}$
Fw-Del-PccdAB	GATTCACCTCTTTTTTACATACTTCAGGTATGCAATTAAGTATACATTCCTCTCTACGCCGGACGCATCGTG	Amplification *aph-parE* cassette from pKD267 to delete P*ccdAB*$_{ST}$
Rv-Del-PccdAB	CTTCCGGAAAGTCGTGAATAGACAAAAAATCGGATCACCTGAAAATAGCCACTGATCAGTGATAAGCTGTC	Amplification *aph-parE* cassette from pKD267 to delete P*ccdAB*$_{ST}$

Name	Primer sequence (5′– 3′)	Used for
PccdAB-5.1	CATATCAGATCCCCCGGAACATC	Amplification P*ccdAB*$_{ST}$ upstream region
PccdAB-3.1	CTTCCGGAAAGTCGTGAATAGACAAAAAATCGGATCACCTGAAAATAGCCATGAAGCAGCGAATTACAGTGACAG	Amplification P*ccdAB*$_{ST}$ upstream region
PccdAB-5.2	AGCAGCTGATAGCTGTCGCTGTCCACTGTCACTGTAATTCGCTGCTTCATGGCTATTTTCAGGTGATCCGATTTTTTG	Amplification P*ccdAB*$_{ST}$ downstream region
PccdAB-3.2	ACCATCATTTTGTGGACCTTTTGG	Amplification P*ccdAB*$_{ST}$ downstream region
Fw-Del-parAB	GTTAGCGTACGCCCTTTCACACGCTTGCGCGCGAACTTGTCCTTTTCTTCTCTCTACGCCGGACGCATCGTG	Amplification *aph-parE* cassette from pKD267 to delete *parAB*
Rv-Del-parAB	GATTGTGGATGTAGTGCCCTCTTCTCCATTTAATCTTCGGTGCCTCCTGTACTGATCAGTGATAAGCTGTC	Amplification *aph-parE* cassette from pKD267 to delete *parAB*
ParAB-5.1	GCCGTCAGAGCTCTGGACAG	Check *parAB* deletion by *aph-parE*
ParAB-3.2	GGCGGAGATCATCCTCAACAAAC	Check *parAB* deletion by *aph-parE*
Fw-Del-spvA	GGGGGAACGGTAATCGCTAACTGTCGGGCAAAGGTATTCAGTGCTTCAAATCTCTACGCCGGACGCATCGTG	Amplification *aph-parE* cassette from pKD267 to insert *aph-parE* cassette in pSLT
Rv-Del-spvA	CCCTGCAGACATTATCAGTCTTCAGGATTTCATTCTGTTTATTTTCAGGAACTGATCAGTGATAAGCTGTC	Amplification *aph-parE* cassette from pKD267 to insert *aph-parE* cassette in pSLT

Name	Primer sequence (5′– 3′)	Used for
SpvA-5.1	CGACCAAGAAACGGGAGACACCAG	Check *aph-parE* insertion in *spvA*
SpvA-3.2	AGCGTTTGAACCAGCACTTCTC	Check *aph-parE* insertion in *spvA*
Fw-Mut-ccdB	CGTAGACTACTGAGCAGCACCTGTTCGCTGACACGCATATCAGATCCCCCTCTCTACGCCGGACGCATCGTG	Amplification *aph-parE* cassette from pKD267 to introduce R99W mutation in $ccdB_{ST}$ in pSLT plasmid
Rv-Mut-ccdB	ATCTCAGCCTCCGGGAGAACGATATCAAAAACGCCATTAACCTGATGTTCACTGATCAGTGATAAGCTGTC	Amplification *aph-parE* cassette from pKD267 to introduce R99W mutation in $ccdB_{ST}$ in pSLT plasmid
CcdB-Mut-5.1	GGAAACCAGCTCGCCGGATGCAAATC	Amplification $ccdB_{ST}$ upstream region
CcdB-Mut-3.1	CGCCATTAACCTGATGTTCTGGGGGATCTGATATGCGTGTC	Amplification $ccdB_{ST}$ upstream region
CcdB-Mut-5.2	CACGCATATCAGATCCCCCAGAACATCAGGTTAATGGCGTTTTTG	Amplification $ccdB_{ST}$ downstream region
CcdB-Mut-3.2	GCAGCGAATTACAGTGACAGTGGAC	Amplification $ccdB_{ST}$ downstream region
PccdAB-5-Eco	CGGGAATTCGTCCTGTAATTCCGTTACG	Cloning $PccdAB_{\mathrm{ST}}$ promoter into pMP220. Introduce a EcoRI restricction site
PccdAB-3-Kpn	CGGGGTACCGATTCACCTCTTTTTTACATACTTCAG	Cloning $PccdAB_{\mathrm{ST}}$ promoter into pMP220. Introduce a KpnI restricction site
CcdB-5′	AGAGTCGCTACCGGCTGTTTGTG	Confirm polycistronic operon
0078-3′	CGAGGGTCTGTGTCCAGGTTTC	Confirm polycistronic operon

Name	Primer sequence (5′– 3′)	Used for
0077-3′	GATGCAAATCCGCAGGTATATCCC	Confirm polycistronic operon
0076-3′	CGTTAATCCGTGCGCCGGTATTC	Confirm polycistronic operon
0075-3′	TAACCAGGCTCAGGCTGAGATTC	Confirm polycistronic operon
Fw-tag-rsdB	AGTTTCAGATGCCGGGTGCTGATGCAGTGGCTATGCTCAAAGGAGGTTCAGACTACAAAGACCATGACGGTG	Tagging rsdB
Rv-tag-rsdB	CCGGATGGCCTGTTGCAGGCGAAAGGCTGAAAAATGCATACACGTCTCTACATATGAATATCCTCCTTAGTTCC	Tagging rsdB
Km-Comp5	CACGATGCGTCCGGCGTAGAG	Check *aph-parE* insertions
Km-Comp3	GACAGCTTATCACTGATCAG	Check *aph-parE* insertions
